# *Helicobacter pylori*-induced adrenomedullin modulates IFN-γ-producing T-cell responses and contributes to gastritis

**DOI:** 10.1038/s41419-020-2391-6

**Published:** 2020-03-17

**Authors:** Hui Kong, Nan You, Han Chen, Yong-sheng Teng, Yu-gang Liu, Yi-pin Lv, Fang-yuan Mao, Ping Cheng, Weisan Chen, Zhuo Zhao, Quan-ming Zou, Gang Guo, Jin-yu Zhang, Yuan Zhuang

**Affiliations:** 1 National Engineering Research Centre of Immunological Products, Department of Microbiology and Biochemical Pharmacy, College of Pharmacy and Laboratory Medicine, Third Military Medical University, Chongqing, China; 2 Department of Blood Transfusion, Daping Hospital of Chinese Army Medical University, Chongqing, China; 3 Department of Hepatobiliary Surgery, Xinqiao Hospital, Third Military Medical University, Chongqing, China; 4 Department of Clinical Laboratory, The 95th Hospital of the Chinese People’s Liberation Army, Putian, Fujian Province China; 5 School of Molecular Science, La Trobe University, Bundoora, Victoria 3085 Australia

**Keywords:** Infection, Chronic inflammation

## Abstract

Adrenomedullin (ADM) is a multifunctional peptide that is expressed by many surface epithelial cells, but its relevance to *Helicobacter pylori (H. pylori)*-induced gastritis is unknown. Here, we found that gastric ADM expression was elevated in gastric mucosa of *H. pylori*-infected patients and mice. In *H. pylori*-infected human gastric mucosa, ADM expression was positively correlated with the degree of gastritis; accordingly, blockade of ADM resulted in decreased inflammation within the gastric mucosa of *H. pylori*-infected mice. During *H. pylori* infection, ADM production was promoted via PI3K–AKT signaling pathway activation by gastric epithelial cells in a *cagA*-dependent manner, and resulted in increased inflammation within the gastric mucosa. This inflammation was characterized by the increased IFN-γ-producing T cells, whose differentiation was induced via the phosphorylation of AKT and STAT3 by ADM derived from gastric epithelial cells. ADM also induced macrophages to produce IL-12, which promoted the IFN-γ-producing T-cell responses, thereby contributing to the development of *H. pylori*-associated gastritis. Accordingly, blockade of IFN-γ or knockout of IFN-γ decreased inflammation within the gastric mucosa of *H. pylori*-infected mice. This study identifies a novel regulatory network involving *H. pylori*, gastric epithelial cells, ADM, macrophages, T cells, and IFN-γ, which collectively exert a pro-inflammatory effect within the gastric microenvironment.

## Introduction

*Helicobacter pylori (H. pylori)* is a Gram-negative bacterium that infects more than half of the world’s population^1^. *H. pylori* is an important factor of chronic gastritis, peptic ulcer, and other digestive system diseases, and has been classified as a class I carcinogen by WHO^2^. During the infection, gastric epithelial cells produce a variety of cytokines that are involved in the inflammatory gastric environment after infection with *H. pylori*^3^. Besides, many immune cells, such as neutrophils, lymphocytes, and plasma cells, are releasing inflammatory factors in the stomach of *H. pylori* infection^4–6^. Inflammatory reaction to *H. pylori* infection shows special characteristics rarely seen in other organs or biological systems, and the mixed acute and chronic inflammatory reactions contribute to *H. pylori*-associated gastritis, and take place simultaneously during *H. pylori* infection^7–9^.

Adrenomedullin (ADM) is a small active hormone that is expressed throughout the gastrointestinal tract^10^. ADM that consists of 52 amino acids is structurally similar to calcitonin gene-related peptide, dextrin, and pituitary^11^. ADM is abundant in the gastrointestinal tract, especially in the neuroendocrine cells of the gastrointestinal mucosa, the intestinal enterochromaffin cells and the chief cells, and the submucosal cells of the colon^12,13^. The widespread distribution of ADM in the gastrointestinal tract provides an anatomical basis for regulating gastrointestinal physiology and pathology. For example, it has been reported that overexpression of ADM in the stomach can inhibit gastric acid secretion^14^. In other studies, ADM protects the mucosa as an endothelial cell growth factor by promoting mucosal healing^15^, and has anti-inflammatory effects in a mouse DSS-induced colitis model^16^. However, the relationship between ADM and gastric inflammation, especially in *H. pylori*-associated gastritis, is presently unknown.

In our study, we have, for the first time, demonstrated a pro-inflammation role of ADM in *H. pylori* infection and *H. pylori*-induced gastritis. ADM is increased in gastric mucosa of both patients and mice infected with *H. pylori*. *H. pylori* induces ADM production from gastric epithelial cells in a *cagA*-dependent manner via activating PI3K–AKT signaling pathway. ADM derived from gastric epithelial cells promotes IFN-γ-producing T cells via the phosphorylation of AKT and STAT3. Besides, ADM also induces macrophages to produce IL-12, which promotes IFN-γ-producing T-cell responses. In vivo, the increased ADM promotes inflammation and IFN-γ-producing T-cell responses in the stomach during *H. pylori* infection, which contributes to *H. pylori*-associated gastritis. These findings provide a novel regulatory network involving ADM within the gastric microenvironment, and a potentially new target of ADM in the treatment of *H. pylori*-associated gastritis.

## Materials and methods

### Ethical statement, patients, and specimens

The gastric biopsy specimens and blood were collected from 70 *H. pylori*-infected and 40 uninfected patients who underwent upper esophagogastroduodenoscopy for dyspeptic symptoms at XinQiao Hospital (Supplementary Table 1). *H. pylori* infection was determined by ^14^C urea breath test and rapid urease test of biopsy specimens taken from the antrum, and subsequently confirmed by real-time PCR for 16s rDNA and serology test for specific anti-*H. pylori* antibodies (Abs) by ELISA (Beier Bioengineering, China). Real-time PCR was also used to distinguish between the *cagA*-postive and *cagA*-negative individuals. For isolation of human primary gastric epithelial cells, fresh non-tumor gastric tissues (at least 5-cm distant from the tumor site) were obtained from gastric cancer patients who underwent surgical resection, and were determined as *H. pylori*-negative individuals as above at the Southwest Hospital. None of these patients had received chemotherapy or radiotherapy before sampling. Individuals with atrophic gastritis, hypochlorhydria, antibiotics treatment, autoimmune disease, infectious diseases, and multi-primary cancer were excluded. The study was approved by the Ethics Committee of XinQiao Hospital and Southwest Hospital of Third Military Medical University. The written informed consent was obtained from each subject.

### Mice

All breeding and experiments were undertaken with review and approval from the Animal Ethical and Experimental Committee of Third Military Medical University. C57BL/6 interferon-γ^−/−^ (IFN-γ^−/−^) mice were kindly provided by Dr. Richard A. Flavell (Yale University). Wild-type C57BL/6 mice were purchased from Laboratory Animal Center of Third Military Medical University. All mice used in the experiments were female and viral Ab free for pathogenic murine viruses, negative for pathogenic bacteria, including *Helicobacter* spp. and parasites, and were maintained under specific pathogen-free (SPF) conditions in a barrier- sustained facility and provided with sterile food and water.

### Antibodies and other reagents

Details are available in Supplementary Table 2.

### Bacterial culture and infection of mice with bacteria

*H. pylori* NCTC 11637 (*cagA* positive) (WT *H. pylori*), *cagA*-knockout mutant *H. pylori* NCTC 11637 (Δ*cagA*), and *H. pylori* 26695 were grown in brain–heart infusion plates containing 10% rabbit blood at 37 °C under microaerophilic conditions. For infecting mouse, bacteria were propagated in Brucella broth with 5% fetal bovine serum (FBS) with gentle shaking at 37 °C under microaerobic conditions. After culture for 1 day, live bacteria were collected and adjusted to 10^9^ CFU/ml. The mice were fasted overnight and orogastrically inoculated twice at a 1-day interval with 3 × 10^8^ CFU bacteria. Age-matched control wild-type mice were mock-inoculated with Brucella broth. Five to seven mice per group per time point were used for the experiments. *H. pylori* infection status and *H. pylori*-induced gastritis in murine experiments were confirmed using real-time PCR of *H. pylori* 16s rDNA and *cagA*, urease biopsy assays, Warthin–Starry staining, and immunohistochemical staining for *H. pylori*, and evaluation of inflammation by hematoxylin and eosin (H&E) staining (data not shown).

### Preparation of rabbit anti-mouse ADM Ab

Polyclone Abs against ADM were produced by ChinaPeptides Co. Ltd. Female New Zealand rabbits received injections at multiple subcutaneous sites with 100 µg of purified peptide emulsified with complete Freund’s adjuvant according to their protocols. Then the rabbits were further immunized at 2-week intervals with 100 µg of ADM emulsified with incomplete Freund’s adjuvant. The anti-sera obtained after the fourth booster injection were screened for anti-ADM activity, and then affinity purified on CNBR-activated sepharose 4b Fast Flow columns.

### In vivo blockade of ADM

One hour before infection with WT *H. pylori*, rabbit anti-mouse ADM Ab (20 µg), or normal rabbit IgG (20 μg) was administered to each mouse via intraperitoneal injection, and the administration was repeated every week until the mice were killed at the indicated time. The dose of ADM Ab was determined on the basis of the data of preliminary experiments in which increasing amounts of ADM Ab (5 μg, 10 μg, 20 μg, and 50 μg per mouse) were used to determine the best dose of ADM Ab that inhibits the expression of IFN-γ in vivo (data not shown).

### In vivo blockade of IFN-γ or IFN-γ administration

One hour before infection with WT *H. pylori*, rabbit anti-mouse IFN-γ mAb (20 µg), or control isotype IgG (20 µg), mouse IFN-γ (20 µg), or equal volume of sterile PBS was administered to each mouse via intraperitoneal injection, and the administration was repeated every week until the mice were killed at the indicated time.

### Evaluation of inflammation and bacterial load

The mice were killed at the indicated time. The stomach was cut open from the greater curvature, and half of the tissue was cut into four parts for RNA, DNA, tissue fixation for H&E staining, and protein extraction, respectively. The degree of inflammation was evaluated in a blinded fashion by two pathologists, and each section was given a score of 0–5 according to the established criteria^17^. DNA of the biopsy specimens was extracted with QIAamp DNA Mini Kit. The density of *H. pylori* colonization was quantified by real-time PCR, detecting *H. pylori*-specific 16s rDNA as previously described^18^, using specific primer and probe (Supplementary Table 3). Expression of 16s rDNA was measured using the TaqMan method. The amount of mouse β2-microglobulin DNA in the same specimen was measured to normalize the data. The density of *H. pylori* in the samples was expressed as the number of bacterial genomes per nanogram of host genomic DNA according to a previous report^19^. Another half of the stomach was used for isolation of single cells. The isolated single cells were collected and analyzed by flow cytometry.

### Isolation of single cells from tissues

Fresh tissues were washed three times with Hank’s solution containing 1% FBS, cut into small pieces, collected in RPMI-1640 containing 1 mg/ml collagenase IV and 10 mg/ml DNase I, and then mechanically dissociated by using the gentle MACS Dissociator (Miltenyi Biotec). Dissociated cells were further incubated for 0.5–1 h at 37 °C under continuous rotation. The cell suspensions were then filtered through a 70-μm cell strainer (BD Labware).

### Human gastric epithelial cell/tissue culture and stimulation

Primary gastric epithelial cells were purified from gastric tissue single-cell suspensions from uninfected donors with a MACS column purification system using anti-human CD326 magnetic beads. The sorted primary gastric epithelial cells were used only when their viability was determined >90%, and their purity was determined >95%. For human gastric epithelial cell lines (AGS cells and HGC-27 cells), 3 × 10^5^ cells per well in a 12-well cell culture plate (for real-time PCR) or 1 × 10^6^ cells per well in a 6-well cell culture plate (for western blot and ELISA) were starved in DMEM (Dulbecco’s Modified Eagle Medium)/F-12 medium supplemented with penicillin (100 U/ml) and streptomycin (100 μg/ml) for 6 h in a humidified environment containing 5% CO_2_ at 37 °C. Then the cells were incubated in antibiotic-free DMEM/F-12 medium supplemented with 10% FBS instead. The cell lines were used when their viability was determined >90%. Human gastric epithelial cell lines, primary gastric epithelial cells, or primary gastric mucosa tissues from uninfected donors were stimulated with WT *H. pylori*, Δ*cagA*, or *H. pylori* 26695 at a multiplicity of infection (MOI) of 100 for 24 h. AGS and HGC-27 cells were also stimulated with WT *H. pylori* or *H. pylori* 26695 at different MOIs (24 h) or at the indicated time points (MOI = 100). For signal pathway inhibition experiments, AGS cells were pretreated with 5 μl (20 μM) SP600125 (a JNK inhibitor), SB203580 (a p38 MAPK inhibitor), AG490 (a JAK inhibitor), or Wortmannin (a PI3K–AKT inhibitor) for 2 h. After coculture, cells were collected for real-time PCR, and western blot, and the culture supernatants were harvested for ELISA or were concentrated for western blot.

### T-cell culture and stimulation

Human T cells were isolated from peripheral blood mononuclear cells (PBMCs) of uninfected donors, and cultured as described previously^20^. Briefly, in a 5-day incubation, bead-purified peripheral CD3^+^ T cells (2 × 10^5^ cells/well in 96-well plates) were labeled with carboxyfluorescein succinimidyl ester (CFSE), and were stimulated with various concentrations of ADM (10–100 nM) in 200 μl of RPMI-1640 medium containing human recombinant (hr) IL-2 (20 IU/ml), anti-CD3 (2 μg/ml), and anti-CD28 (1 μg/ml) antibodies. For signal pathway inhibition experiments, T cells were pretreated with 5 μl (20 μM) SP600125 (a JNK inhibitor), SB203580 (a p38 MAPK inhibitor), U0126 (an MEK-1 and MEK-2 inhibitor), Wortmannin (a PI3K–AKT inhibitor), or FLLL32 (a STAT3 phosphorylation inhibitor) for 2 h. In other cases, T cells were stimulated with the culture supernatants containing human recombinant (hr) IL-2 (20 IU/ml), anti-CD3 (2 μg/ml), and anti-CD28 (1 μg/ml) antibodies from human primary gastric epithelial cells infected with WT *H. pylori* (MOI = 100, 24 h). In some cases, ADM fragment 22–52 (an ADM receptor antagonist) (1 μg/ml) was added into the culture supernatants, and incubated for 2 h before stimulation. After 5-day coculture, cells were collected for flow cytometry and western blot, and the supernatants were harvested for ELISA.

### Macrophage culture and stimulation

Human CD14^+^ cells were isolated from PBMCs of uninfected donors, and cultured as described previously^2^. To get macrophages, bead-purified CD14^+^ cells (2 × 10^4^ cells/well in 96-well plates) were stimulated with GM-CSF (100 ng/ml) for 5 days. Macrophages were stimulated with ADM (100 nM) for 24 h, and the cells were collected for real-time PCR, and the supernatants were harvested for ELISA. In other cases, after stimulation with ADM (100 nM) for 24 h, macrophages were then cocultured with CFSE-labeled T cells (2 × 10^5^ cells/well in 96-well plates) in new complete RPMI-1640 medium containing rhIL-2 (20 IU/ml), anti-CD3 (2 μg/ml), and anti-CD28 (1 μg/ml) antibodies. In some cases, anti-human IL-12 Ab (20 μg/ml) or control IgG was added into the coculture supernatants. After 5-day coculture, T cells were collected for flow cytometry, and the supernatants were harvested for ELISA.

### Immunohistochemistry

Gastric tissue samples were fixed with paraformaldehyde and embedded with paraffin, and then were cut into 5-µm sections. For immunohistochemical staining, goat anti-mouse ADM Abs were used as primary Ab, and rabbit anti-goat HRP as the second Ab; then sections were stained by DAB reagent. After that, all the sections were counterstained with hematoxylin and reviewed using a microscope (Nikon Eclipse 80i, Nikon).

### Immunofluorescence

Paraformaldehyde-fixed sections of gastric tissues were washed in PBS and blocked for 30 min with 20% goat serum in PBS, and stained for ADM, CD326, H^+^/K^+^ ATPase, pepsinogen II, CD3, IFN-γ, CD68, CD45, CD20, CD57, CD11c, F4/80, and RAMP2. Slides were examined with a confocal fluorescence microscope (TCS SP8, Leica).

### Real-time PCR

DNA of the biopsy specimens was extracted with QIAamp DNA Mini Kit, and RNA of biopsy specimens and cultured cells was extracted with TRIzol reagent. The RNA samples were reverse-transcribed to cDNA with PrimeScript^TM^ RT reagent Kit. Real-time PCR was performed on the IQ5 (Bio-Rad) with the Real-time PCR Master Mix according to the manufacturer’s specifications. The expression of 16s rDNA, *cagA*, ADM, IFN-γ, and IL-12p35 genes was measured using the TaqMan and/or SYBR green method with the relevant primers (Supplementary Table 3). For mice, mouse β2-microglobulin mRNA level served as a normalizer, and its level in the stomach of uninfected or WT mice served as a calibrator. For humans, human glyceraldehyde-3-phosphate dehydrogenase (GAPDH) mRNA level served as a normalizer, and its level in the unstimulated cells or stomach of uninfected donors served as a calibrator. The relative gene expression was expressed as fold change of the relevant mRNA calculated by the ΔΔCt method.

### Enzyme-linked immunosorbent assay

Human and mouse gastric tissues from specimens were collected, homogenized in 1 ml of sterile Protein Extraction Reagent, and centrifuged. Tissue supernatants were collected for ELISA. Concentrations of IFN-γ in the tissue supernatants, concentrations of IFN-γ or IL-12 in T-cell culture supernatants, or macrophage culture supernatants were determined using ELISA kits according to the manufacturer’s instructions.

### Western blot analysis

Western blot assays were performed on 10–15% SDS-PAGE gel-transferred PDF membranes using equivalent amounts of cell or tissue lysate proteins of samples, or the concentrated culture supernatants. Three-percent BSA was used for blocking the PDF membranes. Human ADM was detected with rabbit anti-ADM Abs; human p-AKT, AKT, p-STAT3, STAT3, and GAPDH were detected with rabbit anti-p-AKT Abs, mouse anti-AKT Abs, rabbit anti-p-STAT3 Abs, rabbit anti-STAT3 Abs, and mouse anti-GAPDH Abs, respectively. This was followed by incubation with HRP-conjugated secondary Abs. Bound proteins were visualized by using SuperSignal^®^ West Dura Extended Duration Substrate kit.

### Flow cytometry

Flow cytometric analysis was performed according to standard protocols. For intracellular cytokine measurements, the cells were stimulated with Leukocyte activation cocktail with BD GolgiPlug for 5 h. Intracellular cytokine staining was performed after fixation and permeabilization using Perm/Wash solution. The cells were analyzed by multicolor flow cytometry with FACSCanto II (BD Biosciences). Data were analyzed with FlowJo software (TreeStar) or FACSDiva software (BD Biosciences).

### Statistical analysis

The results are expressed as mean ± SEM. Student *t* test was generally used to analyze the differences between two groups, but when the variances differed, the Spearman rank-correlation analysis was used. Inflammation score data were analyzed by the Mann–Whitney *U* test. Correlations between parameters were assessed using Pearson correlation analysis and linear regression analysis, as appropriate. SPSS statistical software (version 13.0) was used for all statistical analyses. All data were analyzed using two-tailed tests, and *P* < 0.05 was considered statistically significant.

## Results

### Adrenomedullin is increased in gastric mucosa of *H. pylori*-infected patients and mice

**Fig. 1:**
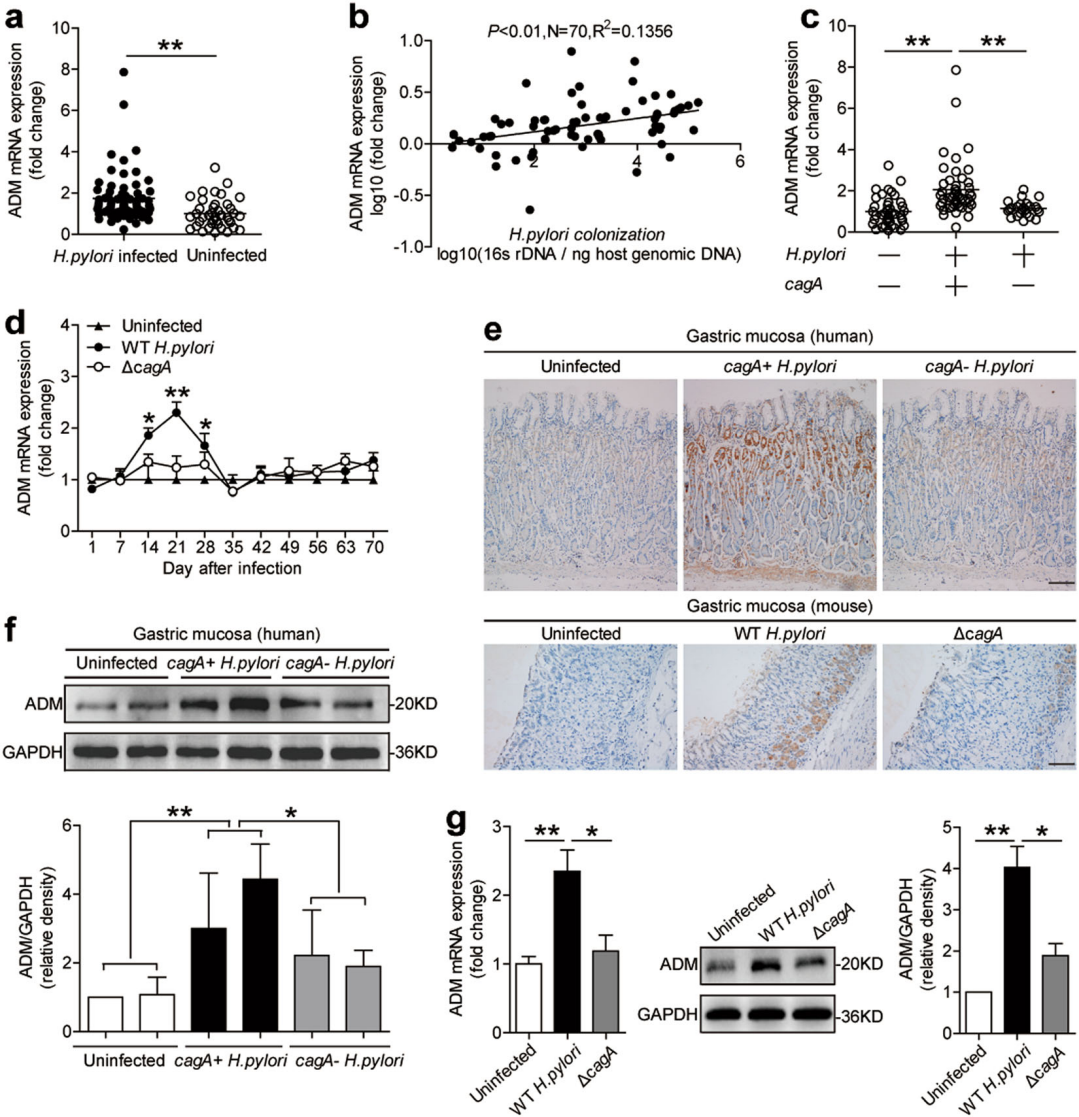
ADM is increased in gastric mucosa of *H. pylori*-infected patients and mice. **a** ADM mRNA expression in gastric mucosa of uninfected donors (*n* = 40) and *H. pylori*-infected patients (*n* = 70) was compared. **b** The correlation between ADM expression and *H. pylori* colonization in gastric mucosa of *cagA*^+^ *H. pylori*-infected patients was analyzed. **c** ADM mRNA expression in gastric mucosa of uninfected donors (*n* = 40), *cagA*^−^ *H. pylori*-infected (*n* = 24), and *cagA*^+^ *H. pylori*-infected (*n* = 46) patients was compared. **d** Dynamic changes of ADM mRNA expression in WT *H. pylori*-infected, Δ*cagA*-infected, and uninfected mice. *n* = 5 per group per time point in **d**. **e** ADM protein in gastric mucosa of uninfected donors, *cagA*^−^ *H. pylori*-infected, and *cagA*^+^ *H. pylori*-infected patients, or in gastric mucosa of WT *H. pylori*-infected, Δ*cagA*-infected, and uninfected mice on day 21 p.i., was analyzed by immunohistochemical staining. Scale bars: 100 µ. **f** ADM protein in gastric mucosa of uninfected donors, *cagA*^–^ *H. pylori*-infected, and *cagA*^+^ *H. pylori*-infected patients was analyzed by western blot and statistically analyzed (*n* = 3). **g** ADM mRNA expression and ADM protein in human primary gastric mucosa from uninfected donors infected with WT *H. pylori* or Δ*cagA* ex vivo, analyzed by real-time PCR and western blot and statistically analyzed (*n* = 3). The results are representative of three independent experiments. The horizontal bars in panels **a** and **c** represent mean values. Each ring or dot in panels **a**–**c** represents one patient or donor. *P < 0.05, and **P < 0.01 for groups connected by horizontal lines compared, or compared with uninfected mice.

To evaluate the potential role of adrenomedullin (ADM) in *H. pylori*-associated pathology, first, we compared the ADM levels in gastric tissues. Notably, compared with uninfected donors, the overall level of ADM mRNA was higher, respectively, in gastric mucosa of *H. pylori*-infected patients (Fig. 1a). Next, ADM expression was positively correlated with *H. pylori* colonization (Fig. 1b), suggesting induction of ADM by *H. pylori*.

The presence of *cagA* is strongly associated with the development of *H. pylori*-associated gastritis^21^. Notably, we found that ADM mRNA expression in *cagA*-positive patients was significantly higher than that in *cagA*-negative individuals (Fig. 1c). Immunohistochemical staining (Fig. 1e) and western blot analysis (Fig. 1f) showed that ADM protein in *cagA*-positive patients was also significantly higher than that in *cagA*-negative individuals. Consistent with our findings in humans, the levels of ADM mRNA (Fig. 1d) and protein (Fig. 1e) were almost only detected in WT *H. pylori*-infected mice, reaching a peak 21 days post infection (p.i.), indicating that a role for *cagA* is involved in the induction of ADM in gastric mucosa during *H. pylori* infection. Finally, we examined the expression of ADM in human primary gastric mucosa stimulated with *H. pylori*, and found that the levels of ADM mRNA and protein in human primary gastric mucosa were significantly increased compared with the samples either not infected or infected with Δ*cagA* (Fig. 1g). Taken together, these findings suggest that ADM is increased in *H. pylori*-infected gastric mucosa of patients and mice.

### Gastric epithelial cells stimulated by *H. pylori* express adrenomedullin

**Fig. 2:**
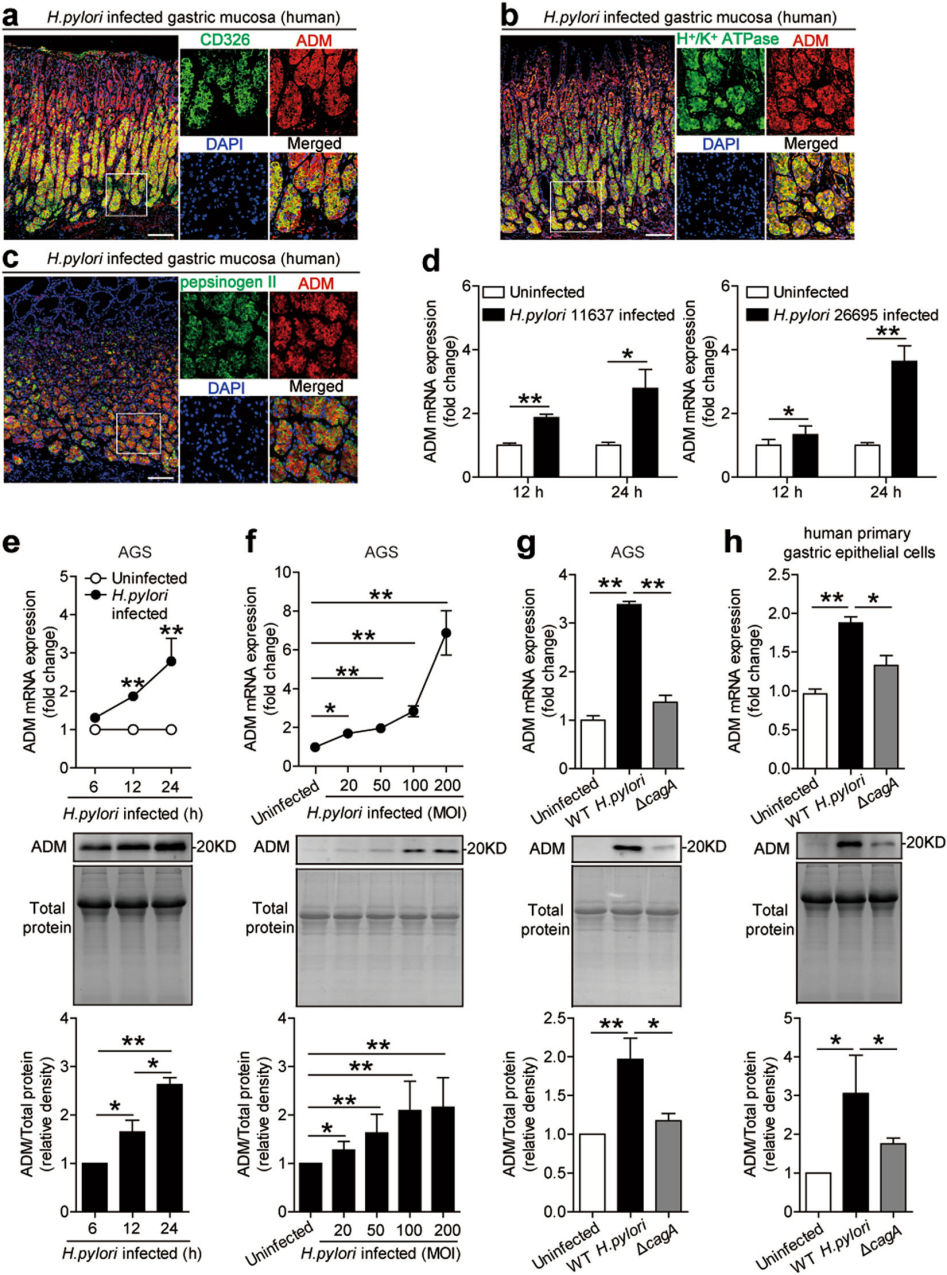
Gastric epithelial cells stimulated by *H. pylori* express ADM. **a** Representative immunofluorescence staining images showing ADM-expressing (red) CD326^+^ gastric epithelial cells (green) in gastric mucosa of *H. pylori*-infected patients. Scale bars: 100 µ. **b** Representative immunofluorescence staining images showing ADM-expressing (red) H^+^/K^+^ ATPase^+^ parietal cells (green) in gastric mucosa of *H. pylori*-infected patients. Scale bars: 100 µ. **c** Representative immunofluorescence staining images showing ADM-expressing (red) pepsinogen II^+^ chief cells (green) in gastric mucosa of *H. pylori*-infected patients. Scale bars: 100 µ. **d** ADM mRNA expression in *H. pylori* 11637-infected, *H. pylori* 26695-infected, and uninfected AGS cells at 12 or 24 h (MOI = 100) was analyzed by real-time PCR (*n* = 3). **e**, **f** ADM mRNA expression and ADM protein in/from WT *H. pylori*-infected and uninfected AGS cells at different time points (MOI = 100) **e** or with different MOI (24 h) **f** were analyzed by real-time PCR and western blot and statistically analyzed (*n* = 3). The results are representative of three independent experiments. Protein loading is shown in the coomassie brilliant blue staining gel. **g**, **h** ADM mRNA expression and ADM protein in/from WT *H. pylori*-infected, Δ*cagA*-infected, and uninfected AGS cells (**g**) or human primary gastric epithelial cells (**h**) (MOI = 100, 24 h) were analyzed by real-time PCR and western blot and statistically analyzed (*n* = 3). The results are representative of three independent experiments. Protein loading is shown in the coomassie brilliant blue staining gel. The supernatants were used to measure the expression of ADM by western blot, and the total cell lysates were used to measure the expression of ADM by real-time PCR. *P < 0.05, and **P < 0.01 for groups connected by horizontal lines compared, or compared with uninfected cells.

Gastric epithelial cells are known to be the first-contacting cell type in gastric mucosa during *H. pylori* infection^22^. We therefore sought to determine whether gastric epithelial cells were responsible for ADM expression during *H. pylori* infection. Interestingly, within gastric mucosa of *H. pylori*-infected donors, ADM was not only expressed in CD326^+^ gastric epithelial cells (Figs. 2a and S1a), but also expressed in H^+^/K^+^ ATPase^+^ parietal cells (Figs. 2b and S1a) and pepsinogen II^+^ chief cells (Figs. 2c and S1a). These data suggest that gastric epithelial cells are the cells that express ADM in gastric mucosa during *H. pylori* infection.

Next, we used two strains of *H. pylori* to infect AGS cells, an immortalized human gastric epithelial cell line, and found that *H. pylori* significantly increased ADM expression (Fig. 2d). Moreover, *H. pylori*-infected AGS cells could increase ADM mRNA expression and ADM protein production in a time-dependent (Fig. 2e) and infection dose-dependent manner (Fig. 2f). Notably, compared with uninfected or the ones infected with Δ*cagA*, WT *H. pylori*-infected AGS cells (Fig. 2g) and human primary gastric epithelial cells (Fig. 2h) also potently increased ADM mRNA expression and ADM protein production. Similar observations were made with another human gastric epithelial cell line HGC-27 cells (Fig. S1b, c). Collectively, these results demonstrate that *H. pylori* infection induces ADM in gastric epithelial cells.

### *H. pylori* stimulates gastric epithelial cells to express adrenomedullin via PI3K–AKT pathway

**Fig. 3:**
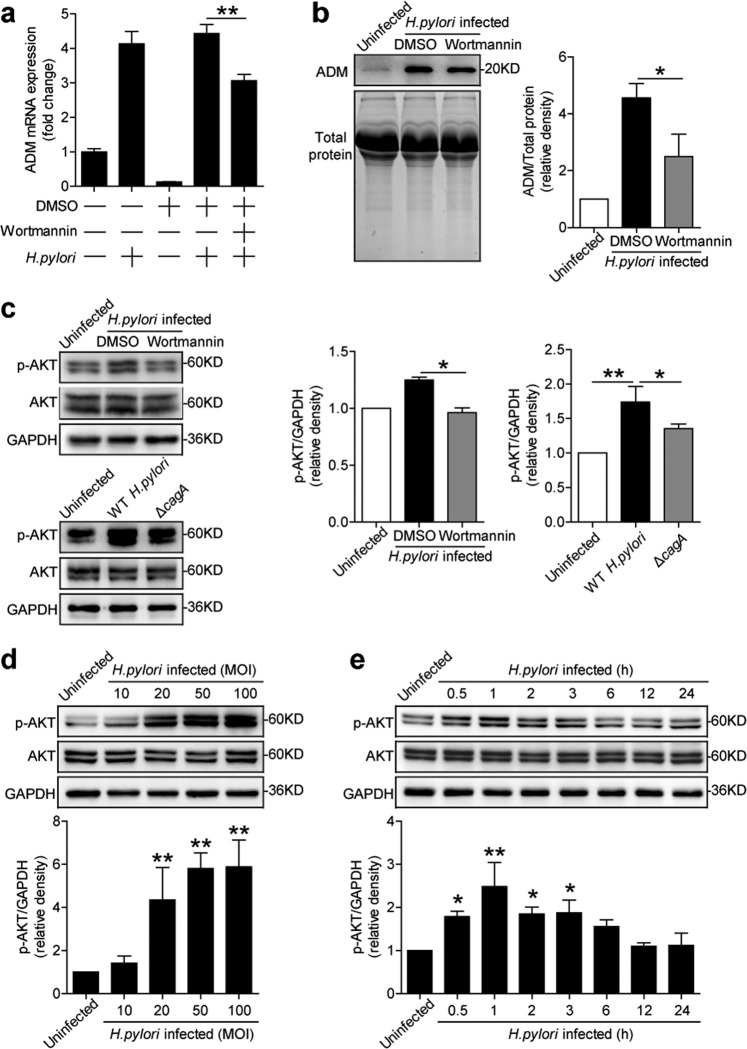
*H. pylori* stimulates gastric epithelial cells to express ADM via PI3K–AKT pathway. **a**, **b** AGS cells were pretreated with Wortmannin (a PI3K–AKT inhibitor) and then stimulated with WT *H. pylori* (MOI = 100) for 24 h. ADM mRNA expression (**a**) and ADM protein (**b**) in/from AGS cells were analyzed by real-time PCR and western blot and statistically analyzed (*n* = 3). The results are representative of three independent experiments. **c** AGS cells were pretreated with Wortmannin (a PI3K–AKT inhibitor) and then stimulated with WT *H. pylori* (MOI = 100) for 1 h, or were infected with WT *H. pylori* or Δ*cagA* (MOI = 100) for 1 h. AKT and p-AKT proteins were analyzed by western blot and statistically analyzed (*n* = 3). The results are representative of three independent experiments. **d**, **e** AGS cells were infected with WT *H. pylori* with different MOI (1 h) (**d**) or at different time points (MOI = 100) (**e**). AKT and p-AKT proteins were analyzed by western blot and statistically analyzed (*n* = 3). The results are representative of three independent experiments. *P < 0.05, and **P < 0.01 for groups connected by horizontal lines compared, or compared with uninfected cells.

To see which signaling pathways might operate in the induction of ADM in gastric epithelial cells, we first pretreated AGS cells with the corresponding inhibitors, and then stimulated AGS cells with *H. pylori*. We found that only blocking PI3K–AKT pathway with Wortmannin effectively suppressed ADM mRNA expression and ADM protein production in/from *H. pylori*-infected gastric epithelial cells (Figs. 3a, b and S2). Furthermore, AKT, a direct PI3K–AKT pathway downstream substrate, was predominantly phosphorylated in AGS cells after stimulation with *H. pylori*, and this was more noticeable when infected with a WT *H. pylori* compared with Δ*cagA* (Fig. 3c), and this phosphorylation was abolished when PI3K–AKT signal transduction pathway was blocked with inhibitor Wortmannin (Fig. 3c). Furthermore, *H.* pylori-induced AKT phosphorylation in AGS cells in dose-dependent (Fig. 3d) as well as in time-dependent manners (Fig. 3e). These data imply that activation of PI3K–AKT signaling pathway is crucial for ADM induction by *H. pylori* in gastric epithelial cells.

### In vivo blockade of adrenomedullin significantly reduced inflammation and IFN-γ-producing T-cell responses in the stomach during *H. pylori* infection

**Fig. 4:**
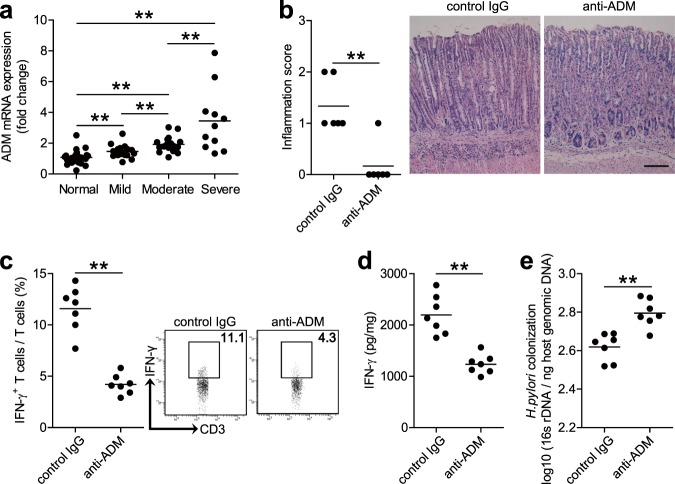
In vivo blockade of ADM significantly reduced inflammation and IFN-γ-producing T-cell responses in the stomach during *H. pylori* infection. **a** ADM mRNA expression in gastric mucosa of *H. pylori*-infected patients with normal gastric histopathology (*n* = 25), or with mild (*n* = 17), moderate (*n* = 17), and severe inflammation (*n* = 11) was compared. **b** Histological scores of inflammation in gastric antra of WT *H. pylori*-infected mice injected with Abs against ADM or the corresponding control IgG on day 21 p.i. were compared. H&E staining, scale bars: 100 µ. **c** The IFN-γ-producing T-cell responses in gastric mucosa of WT *H. pylori*-infected mice injected with Abs against ADM or the corresponding control IgG on day 21 p.i. were compared. **d** The IFN-γ production in gastric mucosa of WT *H. pylori*-infected mice injected with Abs against ADM or the corresponding control IgG on day 21 p.i. was compared. **e** The bacterial colonization in gastric mucosa of WT *H. pylori*-infected mice injected with Abs against ADM or the corresponding control IgG on day 21 p.i. was compared. The horizontal bars in panels **a**–**e** represent mean values. Each dot in panels **a**–**e** represents one patient or mouse. **P < 0.01 for groups connected by horizontal lines compared.

To understand the possible biological effects of ADM induction during *H. pylori* infection, we compared ADM expression within the gastric mucosa with the severity of gastritis observed in patients infected with *H. pylori*. Notably, higher ADM expression was strongly associated with more severe gastritis (Fig. 4a). This led us to hypothesize that ADM might exert pro-inflammatory effects during *H. pylori* infection and thus contribute to gastritis.

To test this hypothesis in vivo, we conducted a series of loss-of-function experiments involving ADM, and evaluated the inflammatory response in gastric mucosa on day 21 p.i. Compared with mice treated with control IgG, mice treated with neutralizing Abs against ADM showed significantly less inflammation in gastric mucosa (Fig. 4b). However, neutralization of ADM did not change the mucosa thickness (Supplementary Fig. 3d). Furthermore, neutralization of ADM significantly reduced the level of IFN-γ-producing T-cell responses (Fig. 4c) and IFN-γ production (Fig. 4d) in gastric mucosa, but had no impact on Ly6G^−^CD11b^+^ monocytes, Ly6G^+^CD11b^+^ neutrophils, CD19^+^ B cells, and NK1.1^+^ natural killer cells (NK cells), IL-4-producing T cells and IL-17-producing T cells in gastric mucosa (Supplementary Fig. 3a), and the numbers of CD3^+^ T cells (Supplementary Fig. 3b). However, neutralization of ADM did not change the level of IFN-γ-producing T-cell responses in Peyer’s patches (Supplementary Fig. 3c). As for the control of bacteria growth by inflammatory immune response^23^, finally, we compared the levels of bacterial colonization in gastric mucosa on day 21 p.i., and found that neutralization of ADM effectively increased *H. pylori* colonization (Fig. 4e). Collectively, these results suggest that ADM has pro-inflammatory effects during *H. pylori* infection in vivo, probably by regulating IFN-γ-producing T-cell responses.

### Adrenomedullin promotes IFN-γ-producing T-cell responses via PI3K–AKT and STAT3 activation

**Fig. 5:**
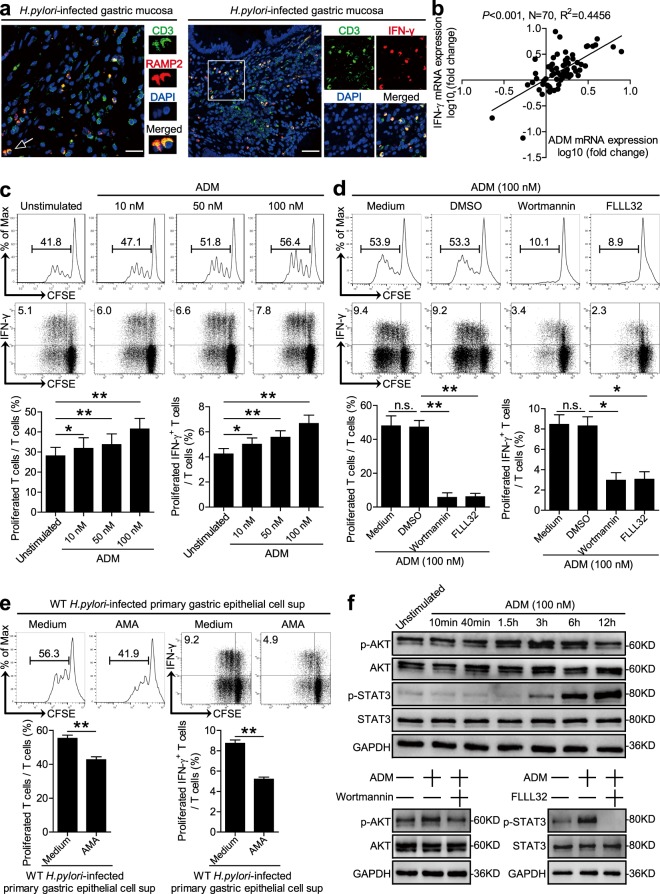
ADM promotes IFN-γ-producing T-cell responses via PI3K–AKT and STAT3 activation. **a** Representative immunofluorescence staining images showing RAMP2-expressing (red) CD3^+^ T cells (green) and IFN-γ-expressing (red) CD3^+^ T cells (green) in gastric mucosa of *H. pylori*-infected patients. Scale bars: 20 µ (left), 100 µ (right). **b** The correlation between ADM expression and IFN-γ expression in gastric mucosa of patients was analyzed. **c**, **d** T cells were stimulated with ADM (10, 50, and 100 nM) (**c**), or pretreated with Wortmannin (a PI3K–AKT inhibitor) and FLLL32 (a STAT3 inhibitor), and then stimulated with ADM (100 nM) (**d**) for 5 days as described in “Materials and methods”. T-cell proliferation and IFN-γ production was assessed by flow cytometry and statistically analyzed (*n* = 3). The results are representative of three independent experiments. **e** T cells were stimulated with the culture supernatants from WT *H. pylori*-infected human primary gastric epithelial cells in the presence or absence of ADM fragment 22–52 (an ADM receptor antagonist) (AMA) for 5 days as described in Methods. T-cell proliferation and IFN-γ production was assessed by flow cytometry and statistically analyzed (*n* = 3). The results are representative of three independent experiments. **f** T cells were stimulated with ADM (100 nM) at different time points, or pretreated with Wortmannin (a PI3K–AKT inhibitor) or FLLL32 (a STAT3 phosphorylation inhibitor), and then stimulated with ADM (100 nM) for 6 h. AKT and p-AKT proteins, and STAT3 and p-STAT3 proteins, were analyzed by western blot. Spearman rank-correlation analysis was used for **c**, *P < 0.05, and **P < 0.01 for groups connected by horizontal lines compared. sup supernatants.

Next, we were therefore interested to know if and how ADM induces IFN-γ-producing T-cell responses. To begin, we found that an IFN-γ-producing T-cell infiltration as well as the expression of ADM receptor domain protein, receptor-modifying protein 2 (RAMP2), merged with CD3 staining on T cells in *H. pylori*-infected human gastric mucosa (Figs. 5a and S4a). Moreover, an increased IFN-γ expression and a significant positive correlation between ADM and IFN-γ expression was found in *H. pylori*-infected human gastric mucosa (Figs. 5b and S4b), altogether suggesting that T cells are a major target of ADM action within the inflamed gastric mucosa during *H. pylori* infection.

To further evaluate the contribution of ADM to the induction of IFN-γ-producing T-cell responses, we stimulated human T cells with ADM, and found that ADM was able to potently induce T-cell proliferation and IFN-γ production in a dose-dependent manner (Figs. 5c and S4d). To see which signaling pathways might operate in the induction of IFN-γ-producing T cells by ADM, we first pretreated human T cells with the corresponding inhibitors, and then stimulated T cells with ADM. We found that blocking PI3K–AKT pathway with Wortmannin, or STAT3 phosphorylation with FLLL32, effectively suppressed T-cell proliferation and IFN-γ production (Figs. 5d and S4c, e). Furthermore, AKT and STAT3 were predominantly phosphorylated in T cells after stimulation with ADM in a time-dependent manner (Figs. 5f and S4f), and this phosphorylation was abolished when PI3K–AKT signal transduction pathway was blocked with Wortmannin, or STAT3 phosphorylation was abolished with FLLL32 (Figs. 5f and S4f). Finally, to ascertain whether ADM from *H. pylori*-stimulated gastric epithelial cells contributes to the induction of IFN-γ-producing T-cell responses, we abolished ADM–ADM receptor interaction with ADM fragment 22–52 (an ADM receptor antagonist) (AMA) on T cells, and then stimulated T cells with the culture supernatants from WT *H. pylori*-stimulated primary gastric epithelial cells. As expected, blocking ADM–ADM receptor interaction effectively inhibited T-cell proliferation and IFN-γ production (Fig. 5e). Taken together, these findings suggest that ADM promotes IFN-γ-producing T-cell responses via activation of PI3K–AKT and STAT3 signaling pathways.

### Adrenomedullin-stimulated macrophages induce IFN-γ-producing T-cell responses

**Fig. 6:**
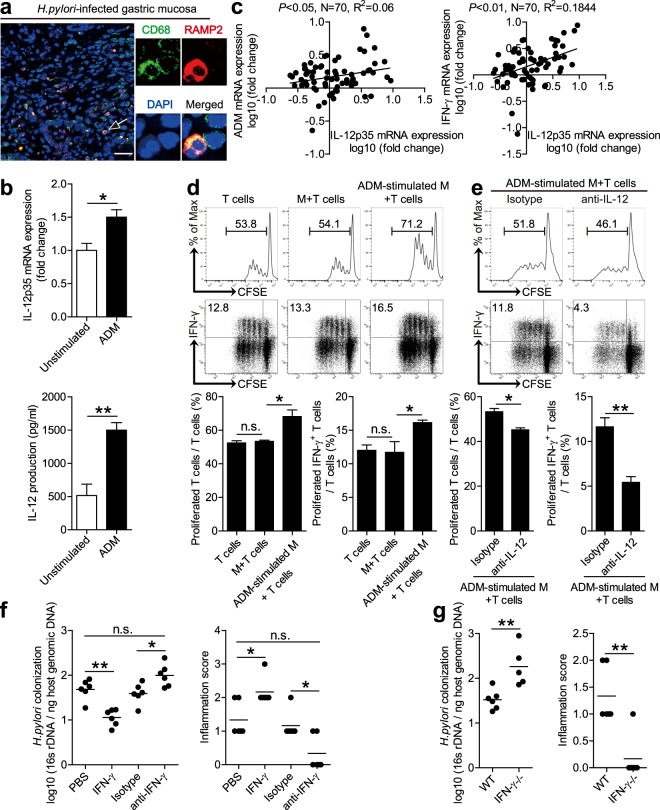
ADM-stimulated macrophages induce IFN-γ-producing T-cell responses. **a** Representative immunofluorescence staining images showing RAMP2-expressing (red) CD68^+^ macrophages (green) in gastric mucosa of *H. pylori*-infected patients. Scale bars: 50 µ. **b** IL-12p35 mRNA expression and IL-12 protein in/from unstimulated or ADM-stimulated human primary macrophages derived from blood monocytes were analyzed by real-time PCR and ELISA (*n* = 3). **c** The correlations between ADM expression and IL-12p35 expression, or IL-12p35 expression and IFN-γ expression in gastric mucosa of patients, were analyzed. **d**, **e** T-cell proliferation and IFN-γ production of T-cell-macrophage coculture was assessed by flow cytometry as described in Methods and statistically analyzed (*n* = 3). **f** Histological scores of inflammation and bacteria colonization in gastric mucosa of WT *H. pylori*-infected mice injected with IFN-γ or PBS control, Abs against IFN-γ or the corresponding control isotype IgG on day 21 p.i. were compared. **g** Histological scores of inflammation and bacteria colonization in gastric mucosa of WT *H. pylori*-infected WT and IFN-γ^−/−^ mice on day 21 p.i. were compared. The horizontal bars in panels **f** and **g** represent mean values. Each dot in panels **c**, **f**, and **g** represents one patient or mouse. *P < 0.05, **P < 0.01, and n.s. *P* > 0.05 for groups connected by horizontal lines compared.

It has previously been shown that IFN-γ-producing T cells are induced by IL-12^24^, and that macrophages are potent producers of IL-12 at sites of bacterial infection^25^. Since macrophages have previously been implicated in *H. pylori* gastritis^26^, we next were interested to learn whether ADM-regulated macrophages would affect IFN-γ-producing T-cell responses via IL-12 during *H. pylori* infection. To begin, we found that an expression of ADM receptor domain protein, RAMP2, merged with CD68 staining on macrophages in *H. pylori*-infected gastric mucosa (Figs. 6a and S5a). Next, ADM was able to potently induce IL-12 expression and production from macrophages (Fig. 6b). Moreover, an increased IL-12 expression and significant positive correlations between ADM and IFN-γ expression, or between IFN-γ and IL-12 expression, were found in *H. pylori*-infected gastric mucosa (Figs. 6c and S5b), Taken together, these findings suggest that macrophages are another major target of ADM action, and may be responsible for the IFN-γ-producing T-cell responses within the inflamed gastric mucosa during *H. pylori* infection.

To further evaluate the contribution of ADM-regulated macrophages to the induction of IFN-γ-producing T-cell responses, we stimulated macrophages with ADM and then cocultured with T cells, and found that ADM-stimulated macrophages were able to potently induce T-cell proliferation and IFN-γ production (Figs. 6d and S5c). To see whether IL-12 from ADM-stimulated macrophages might operate in the induction of IFN-γ-producing T cells, we added with neutralizing antibodies against IL-12 in the coculture system above, and found that blocking IL-12 effectively suppressed T-cell proliferation and IFN-γ production induced by ADM-stimulated macrophages (Figs. 6e and S5d). Taken together, these findings suggest that ADM-stimulated macrophages promote IFN-γ-producing T-cell responses via IL-12.

Finally, we conducted a series of loss- and gain-of-function in vivo experiments involving IFN-γ, and evaluated the inflammatory response and bacterial colonization in gastric mucosa on day 21 p.i. Compared with mice treated with control isotype IgG, mice treated with neutralizing antibodies against IFN-γ showed significantly less inflammation and higher *H. pylori* colonization in gastric mucosa (Fig. 6f). Conversely, injection of IFN-γ significantly increased inflammation and reduced *H. pylori* colonization in gastric mucosa (Fig. 6f). Similarly, compared with WT mice, IFN-γ^−/−^ mice showed significantly less inflammation and higher *H. pylori* colonization in gastric mucosa (Fig. 6g). These suggest that IFN-γ contributes to inflammation during *H. pylori* infection. Collectively, these results suggest that ADM-stimulated macrophages promote IFN-γ-producing T-cell responses, and IFN-γ contributes to inflammation during *H. pylori* infection.

## Discussion

More than 50% of the world’s population infects *H. pylori* in their upper gastrointestinal tracts, which is more common in developing countries than Western countries^1^. *H. pylori* infection is a threat to human health, for example, the long-term colonization of *H. pylori* in the stomach can change the pH of the stomach, promoting chronic gastritis, gastric ulcers, and even gastric cancer^2^. However, until now, the mechanism of *H. pylori*-associated chronic gastritis remains unclear, and it is believed that the interplays between host and bacterial virulence factors^21,27^, such as *vacA*^15,28,29^, especially a major virulence factor *cagA* that can be injected into the host cell by Type IV Secretion System (T4SS)^30^, and that will be phosphorylated after entering the host gastric epithelial cells^31^, and the following persistent inflammation, are likely the underlying causes.

**Fig. 7:**
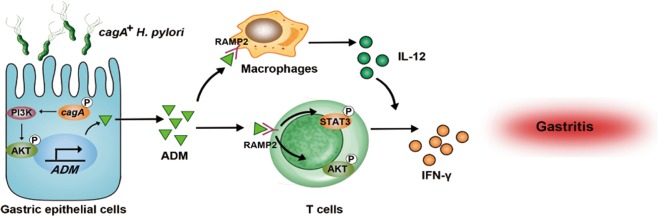
A proposed model of cross-talk among *H. pylori*, gastric epithelial cells, ADM, and macrophages, leading to IFN-γ-producing T-cell responses and ADM–IFN-γ-mediated pro-inflammation in gastric mucosa during *H. pylori* infection. *H. pylori* stimulates gastric epithelial cells to secrete ADM via PI3K–AKT signaling pathway activation in a *cagA*-dependent manner. Release of ADM induces the differentiation of IFN-γ-producing T cells via the phosphorylation of AKT and STAT3. On the other hand, ADM stimulates macrophages to produce IL-12, which promotes the IFN-γ-producing T-cell responses. Increased ADM and IFN-γ exert a pro-inflammatory effect within the gastric microenvironment, which collectively contributes to gastritis during *H. pylori* infection.

In this study, we demonstrated a multistep model of inflammation during *H. pylori* infection within the gastric mucosa involving interactions among *H. pylori*, gastric epithelial cells, T cells, and macrophages via ADM (Fig. 7). In vivo and in vitro, we established that *H. pylori*-associated virulence factor *cagA* was essential to inducing ADM expression in gastric epithelial cells, which in turn promoted gastric inflammation, CD3^+^ T-cell proliferation, and IFN-γ production. Moreover, the increased ADM induced macrophages to secrete IL-12, which also promoted IFN-γ-producing T-cell responses. To our knowledge, this study is the first time to demonstrate the pro-inflammatory role of ADM and its association with macrophages and T cells in *H. pylori*-induced gastritis.

ADM is a biologically active peptide that was isolated in 1993 by Kitamura et al.^32^ from human pheochromocytoma. It was discovered that ADM was able to activate platelet adenylate cyclase and exerted a long-lasting hypotensive effect in rats^32^. The expression of ADM is related to many factors, for example, inflammatory factors IL-1β and TNF-α induce ADM expression in some cell types^33^. Some studies showed that plasmatic ADM levels were raised in many infectious diseases, particularly in sepsis^34^, suggesting an inflammatory role of this hormone. In regard to the digestive system, several studies have demonstrated that ADM levels rise in infected gastrointestinal tissues. For example, examination of ADM expression in gastrointestinal epithelium revealed higher mRNA levels in paratuberculosis-infected versus uninfected cows^35^. It has been reported that infection of *H. pylori* would upregulate the expression of ADM in gastric epithelial cells in vitro^36^. In the urinary system, ADM exerted different roles in primary and cancerous epithelial cells. ADM ameliorated the epithelial-to-mesenchymal transition in human proximal tubular epithelial cells^37^; however, ADM promoted renal cell carcinoma angiogenesis^38^. In our study, first we validated the previous results^36^ in vitro; besides, we confirmed the increased expression of ADM in gastric tissues from 70 *H. pylori*-infected patients, as well as in the mouse models infected with *H. pylori* in vivo. Moreover, we noticed that the expression of ADM was increased during *H. pylori* infection, but the effect was so transient in mice (Fig. 1d, 2–4 weeks). It may be due to multiple factors, one possibility is the loss of function of the type IV secretion system^39,40^, and also may be due to the negative feedback regulation of ADM expression by neurohormones^41^, or the balance between synthesis and metabolism of ADM, which has a very short half-life and easily adheres to surfaces^42^.

ADM can activate a great variety of signal transduction pathways, depending on the species, organ, tissue, and cell type. However, there are three main signaling pathways by which ADM exerts its actions: cAMP, phosphoinositide 3-kinase (PI3K)–AKT, and mitogen-activated protein kinase (MAPK)–extracellular signal-regulated protein kinase (ERK)^32,43,44^. All signal mechanisms in which ADM is involved are the basis of this peptide’s extensive repertoire of biological functions, such as vasodilation, cellular proliferation, apoptosis modulation, or inflammatory regulation^44–47^. It has been reported that ADM can promote the proliferation of cells, such as cardiomyocytes and vascular endothelial cells, via signaling pathways, such as PI3K–AKT and MAPK, by acting on ADM receptor 1 (ADMR1) and ADMR2, and participate in the construction of blood vessels. Simultaneously, studies have shown that ADM receptors are expressed on T cells^48^. However, the direct effect of ADM on T cells in the gastrointestinal tract has not been reported. Our results for the first time show that, during *H. pylori* infection, gastric epithelial cell-derived ADM can directly promote the proliferation of T cells and the production of IFN-γ from T cells through PI3K–AKT and STAT3 signaling pathways. The significance of this finding is to further understand the role of ADM in the alteration of the immune environment in the *H. pylori*-infected stomach.

In our study, we found that after blocking ADM during *H. pylori* infection, gastric inflammation decreased in vivo. However, some studies reported that ADM showed potent anti-inflammatory activity by downregulating the production of a wide panel of inflammatory mediators, such as IL-10 and TNF-α, by microglia and DCs^49^. We guess that ADM may exert different roles by acting on different types of target cells in different diseases. Here, we found that ADM from *H. pylori*-infected gastric epithelial cells directly promoted T-cell proliferation and IFN-γ production. Besides, we also found that ADM indirectly acted on T cells by IL-12 derived by ADM-stimulated macrophages, which resembles the data on IL-1β secretion in synovial inflammation^50^. It will be interesting to see which cell type plays a more important role. It is clear that a mixed acute and chronic inflammatory reaction takes place in *H. pylori*-induced inflammatory environments, where multifarious immune cell types infiltrate and play complicated roles^16,17,51,52^. In a recent report, TLR9^−/−^ mice were found to show increased signs of gastritis upon *H. pylori* infection^53^. This report supports our findings that ADM may play a role in promoting inflammation by directly or indirectly acting on immune cells during *H. pylori* infection. Moreover, we suspect that the inflammatory IFN-γ-producing T-cell responses as well as ADM itself can control *H. pylori* colonization to some extent. However, these responses could not clear *H. pylori* completely due to several reasons inducing the impaired host defense^54^, which contributes to the chronic *H. pylori* infection in gastric mucosa. In this study, the concentration of ADM and the MOI of *H. pylori* in the stimulation of gastric epithelial cells might be both higher than the actual in vivo, and could not fully imitate the model in vivo, which needs to be further studied.

To sum up, our findings provide a novel regulatory network involving *H. pylori*, gastric epithelial cells, ADM, macrophages, T cells, and IFN-γ in *H. pylori*-induced gastritis. Given the notable relationship between the level of ADM and the severity of gastric inflammation observed in *H. pylori*-infected patients, it is possible that ADM might serve as a novel diagnostic and prognostic biomarker for *H. pylori-*associated gastritis. Future clinical studies are necessary to investigate and verify the ADM-associated mechanisms in humans, which may lead to the application of novel pharmacologic approaches to resist this gastric inflammation.

## Supplementary information

Supplementary Table 1

Supplementary Table 2

Supplementary Table 3

Supplementary Figure 1

Supplementary Figure 2

Supplementary Figure 3

Supplementary Figure 4

Supplementary Figure 5

Supplementary Figure Legends
