# Supplementary material for: Helicobacter pylori-induced adrenomedullin modulates IFN-γ-producing T-cell responses and contributes to gastritis
Source: Cell Death Dis. 2020 Mar 17;11(3):189. doi: 10.1038/s41419-020-2391-6 (PMC7078296; doi:10.1038/s41419-020-2391-6)
Supplement: Supplementary file 1 — Supplementary Table 1 [file 41419_2020_2391_MOESM1_ESM.doc]

**Supplementary Table 1.** Clinical characteristics of patients

| Variables | *H. pylori-*infected | Uninfected |
| --- | --- | --- |
| Age (median, range)  Sex (male/female) | (46 year, 22-71 years)  42/28 | (47 year, 19-66 years)  21/19 |

Exclusion criteria were: previous treatment for *H. pylori*-infection, use of inhibitors of acid secretion and/or antibiotics during the 2 months before the study, use of anticoagulant drugs in the last week, gastrointestinal malignancy, severe concomitant cardiovascular, respiratory or endocrine diseases, clinically significant renal or hepatic disease, haematological disorders, previous gastro-oesophageal surgery, history of allergy to any of the drug used in the study, pregnancy or lactation, alcohol abuse, drug addiction, severe neurological or psychiatric disorders, and long-term use of corticosteroids or anti-inflammatory drugs.
