# Supplementary material for: Helicobacter pylori-induced adrenomedullin modulates IFN-γ-producing T-cell responses and contributes to gastritis
Source: Cell Death Dis. 2020 Mar 17;11(3):189. doi: 10.1038/s41419-020-2391-6 (PMC7078296; doi:10.1038/s41419-020-2391-6)
Supplement: Supplementary file 2 — Supplementary Table 2 [file 41419_2020_2391_MOESM2_ESM.doc]

**Supplementary Table 2.** Antibodies and other reagents

| Antibodies and reagents | Manufacturers |  |
| --- | --- | --- |
| Antibodies for flow cytometry |  |  |
| anti-mouse CD45-PE-Cy7 | Biolegend |  |
| anti-mouse IL-17A-PE | Biolegend |  |
| anti-mouse CD11b-PerCP-Cy5.5 | Biolegend |  |
| anti-mouse Ly6G-FITC | Biolegend |  |
| anti-mouse NK1.1-PE | Biolegend |  |
| anti-mouse CD19-APC-Cy7 | Biolegend |  |
| anti-mouse IL-4-APC-Cy7 | Biolegend |  |
| anti-mouse CD3-APC | Biolegend |  |
| anti-mouse IFN-γ-FITC | Biolegend |  |
| anti-human CD3-APC-H7 | BD Pharmingen |  |
| anti-human IFN-γ-PE-Cy7 | BD Pharmingen |  |
| Antibodies for immunohistochemical staining |  |  |
| rabbit anti-human adrenomedullin | Abcam |  |
| goat anti-mouse adrenomedullin | Santa Cruze |  |
| horseradish peroxidase anti-rabbit IgG | Zhongshan Biotechnology |  |
| Antibodies for immunofluorescence  rabbit anti-human adrenomedullin  mouse anti-human CD3  mouse anti-human pepsinogen II  mouse anti-human CD326  mouse anti-human H^+^/K^+^ ATPase  mouse anti-human CD68  rabbit anti-human RAMP2  rabbit anti-human CD45  rabbit anti-human CD20  mouse anti-human CD57  rabbit anti-human CD11c  sheep anti-mouse RAMP2  rabbit anti-mouse F4/80  rabbit anti-human RAMP2  goat anti-human IFN-γ  goat anti-rabbit-TRITC  goat anti-rabbit-FITC  goat anti-mouse-TRITC  goat anti-mouse-FITC  rabbit anti-goat-TRITC  rabbit anti-sheep-TRITC | Abcam  Abcam  Santa Cruz  Abcam  Abcam  Abcam  Invitrogen  Abcam  Abcam  Abcam  Abcam  Abcam  Abcam  Abcam  R&D Systems  Zhongshan Biotechnology  Zhongshan Biotechnology  Zhongshan Biotechnology  Zhongshan Biotechnology  Zhongshan Biotechnology  Zhongshan Biotechnology |  |
| Antibodies for neutralizing and blocking |  |  |
| anti-mouse IFN-γ (Rat IgG1)  Rat IgG1 Isotype Control | Biolegend  Biolegend |  |
| anti-human IL-12 (Goat IgG)  Goat IgG Control | R&D Systems  R&D Systems |  |
| Antibodies for western blot |  |  |
| rabbit anti-human adrenomedullin | Abcam | |
| rabbit anti-human AKT(Ser473) | Cell signaling technology |  |
| rabbit anti-human p-AKT(Ser473)  rabbit anti-human STAT3(Tyr705)  mouse anti-human p-STAT3(Tyr705)  rabbit anti-human/mouse GAPDH  rabbit anti-human/mouse GAPDH | Cell signaling technology  Cell signaling technology  Cell signaling technology  Beijing Ray Antibody Biotech  Abcam |  |
| ELISA kits |  |  |
| human IFN-γ  human IL-12  mouse IFN-γ | Biolegend  Biolegend  Biolegend |  |
| Reagents for signaling pathways inhibition |  |  |
| STAT3 inhibitor FLLL32 | Medco bioscience |  |
| JAK inhibitor AG490 | Merk Millipore |  |
| JNK inhibitor SP600125 | Calbiochem |  |
| MAPK inhibitor SB203580 | Calbiochem |  |
| PI3K-AKT inhibitor Wortmannin | Merk Millipore |  |
| Human CD326 microbeads | Milteniy Biotec |  |
| Human CD3 microbeads | Milteniy Biotec |  |
| Mouse CD326 microbeads  Human CD14 microbeads  Purified anti-CD3 and anti-CD28 antibodies  ADM fragment 22-52 (an ADM receptor antagonist) (AMA) | Milteniy Biotec  StemCell Technologies  Biolegend  Sigma-Aldrich |  |
| Collagenase IV | Gibco |  |
| DNase I | Sigma-Aldrich |  |
| DMSO | Sigma-Aldrich |  |
| Protein Extraction Reagent | Pierce |  |
| SuperSignal® West Dura Extended Duration Substrate kit | Thermo |  |
| Fetal bovine serum (FBS) | Gibco |  |
| Penicillin/Streptomycin | Gibco |  |
| RPMI-1640  DMEM/F12 (1:1) | Hyclone  Hyclone |  |
| Ficoll-Paque Plus | GE Healthcare |  |
| lyses solution  Leukocyte activation cooktail  GolgiPlug  TRIzol reagent | TIANGEN  BD Pharmingen  BD Pharmingen  Invitrogen |  |
| QIAamp DNA Mini Kit | QIAGEN |  |
| PrimeScriptTM RT reagent Kit | TaKaRa |  |
| Real-time PCR Master Mix | Toyobo |  |
| All recombinant human/mouse cytokines and chemokines | PeproTech |  |

APC-Cy7, allophycocyanin-cyanin 7; PE-Cy7, phycoerythrin-cyanin 7; FITC, Fluorescein isothiocyanate; PE, phycoerythrin; PerCP-Cy5.5, peridin chlorophyl protein-cyanin 5.5; APC, allophycocyanin; IL, interleukin; IFN, interferon.
