# Supplementary material for: Helicobacter pylori-induced adrenomedullin modulates IFN-γ-producing T-cell responses and contributes to gastritis
Source: Cell Death Dis. 2020 Mar 17;11(3):189. doi: 10.1038/s41419-020-2391-6 (PMC7078296; doi:10.1038/s41419-020-2391-6)
Supplement: Supplementary file 3 — Supplementary Table 3 [file 41419_2020_2391_MOESM3_ESM.doc]

**Supplementary Table 3. Primer and probe sequences for real-time PCR analysis**

| Gene | Primer or probe | Sequence 5′→3′ |
| --- | --- | --- |
| *H. pylori* 16s rDNA | forward | TTTGTTAGAGAAGATAATGACGGTATCTAAC |
|  | reverse | CATAGGATTTCACACCTGACTGACTATC |
|  | probe | CGTGCCAGCAGCCGCGGT |
| Mouse β2-microglobulin | forward | CCTGCAGAGTTAAGCATGCCAG |
|  | reverse | TGCTTGATCACATGTCTCGATCC |
|  | probe | TGGCCGAGCCCAAGACCGTCTAC |
| *H. pylori* *cagA* | forward | GAGTCATAATGGCATAGAACCTGAA |
|  | reverse | TTGTGCAAGAAATTCCATGAAA |
| Human adrenomedullin | forward | GGAAGAGGGAACTGCGGATGT |
|  | reverse | GGCATCCGGACTGCTGTCT |
| Human IL-12 p35 | forward | GTGTGGAGGCTGGTTAGCAC |
|  | reverse | TGCCCTTGACCTCTGTCACT |
| Human IFN-γ | forward | TCGCCAGCAGCTAAAACAGG |
|  | reverse | TTGCAGGCAGGACAACCATT |
| Human GAPDH | forward | ACCCAGAAGACTGTGGATGG |
|  | reverse | CAGTGAGCTTCCCGTTCAG |
| Mouse β-actin | forward | AGTGTGACGTTGACATCCGT |
|  | reverse | GCAGCTCAGTAACAGTCCGC |
| Mouse adrenomedullin | forward | TTCGCAGTTCCGAAAGAAGT |
|  | reverse | CCAGTTGTGTTCTGCTCGTC |

For the probes, a FAM fluorescent reporter is coupled to the 5' end, and a TAMRA quencher is coupled to the 3' end.
