# Supplementary figures and images for: Helicobacter pylori-induced adrenomedullin modulates IFN-γ-producing T-cell responses and contributes to gastritis
Source: Cell Death Dis. 2020 Mar 17;11(3):189. doi: 10.1038/s41419-020-2391-6 (PMC7078296; doi:10.1038/s41419-020-2391-6)

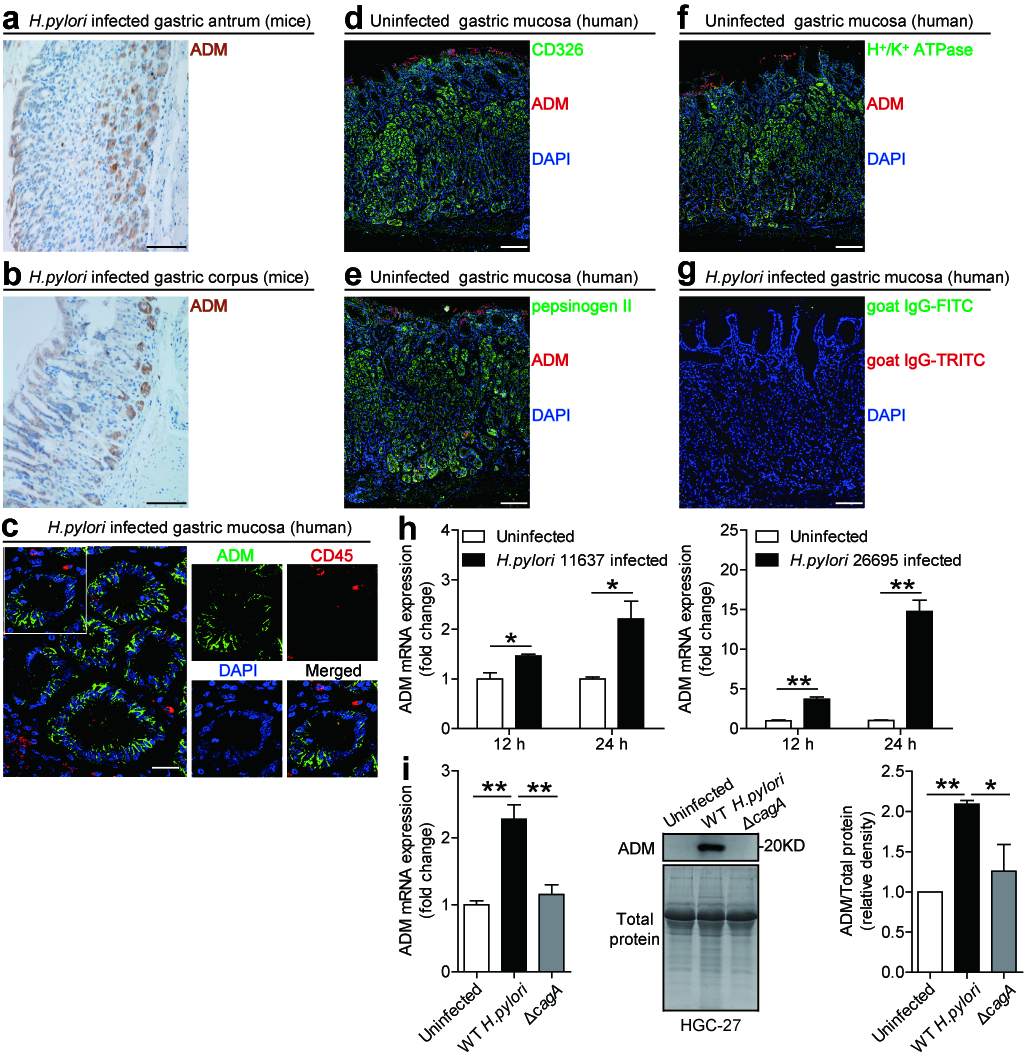

Supplement: Supplementary file 4 — Supplementary Figure 1 [file 41419_2020_2391_MOESM4_ESM.tif]

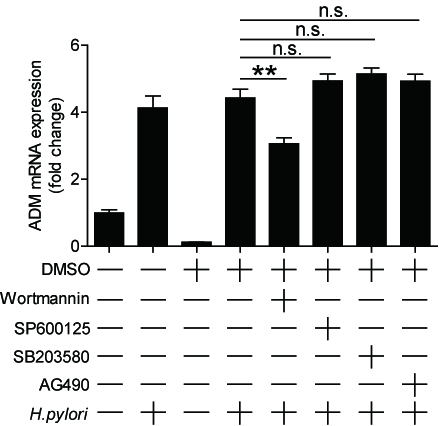

Supplement: Supplementary file 5 — Supplementary Figure 2 [file 41419_2020_2391_MOESM5_ESM.tif]

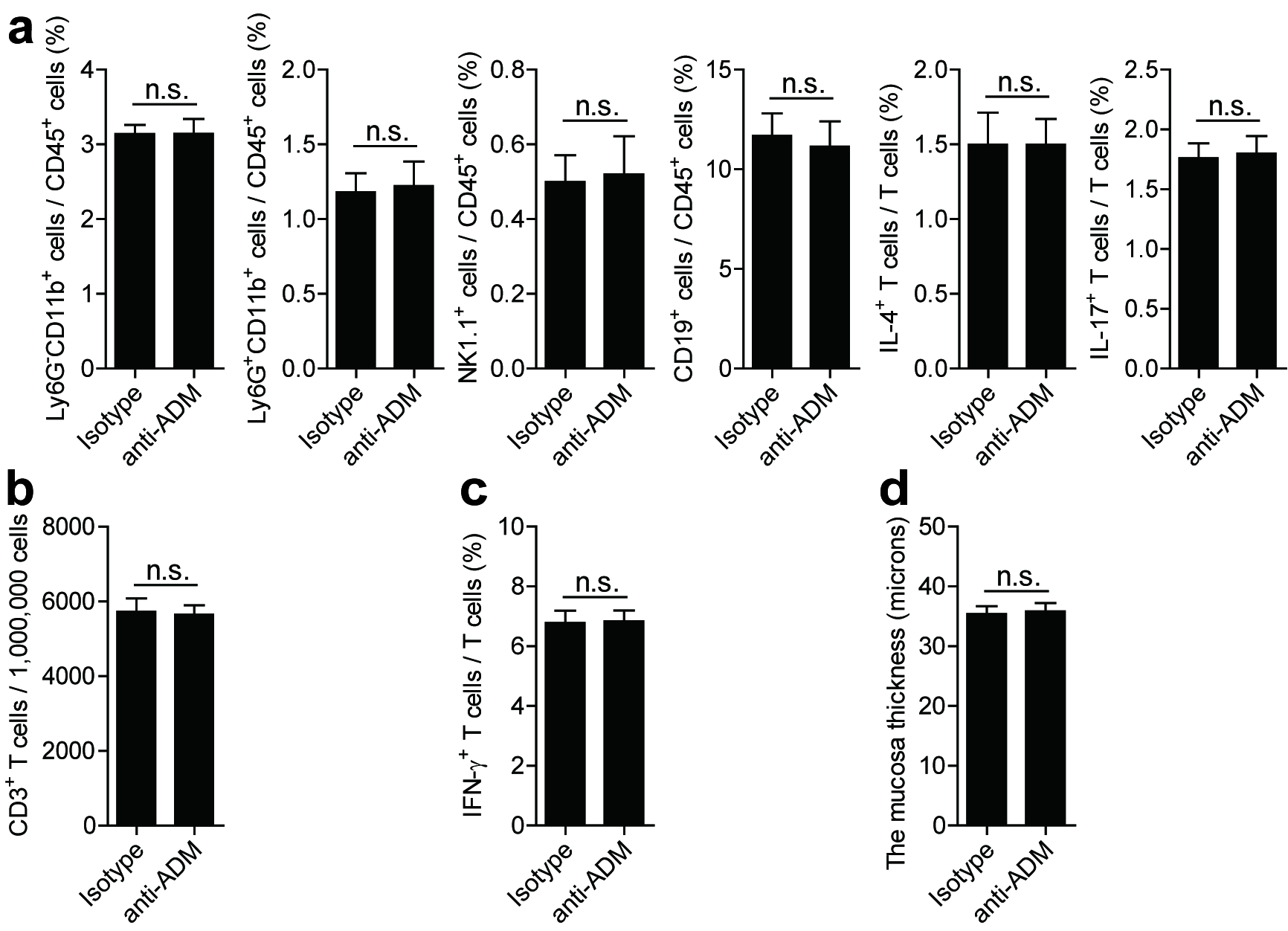

Supplement: Supplementary file 6 — Supplementary Figure 3 [file 41419_2020_2391_MOESM6_ESM.tif]

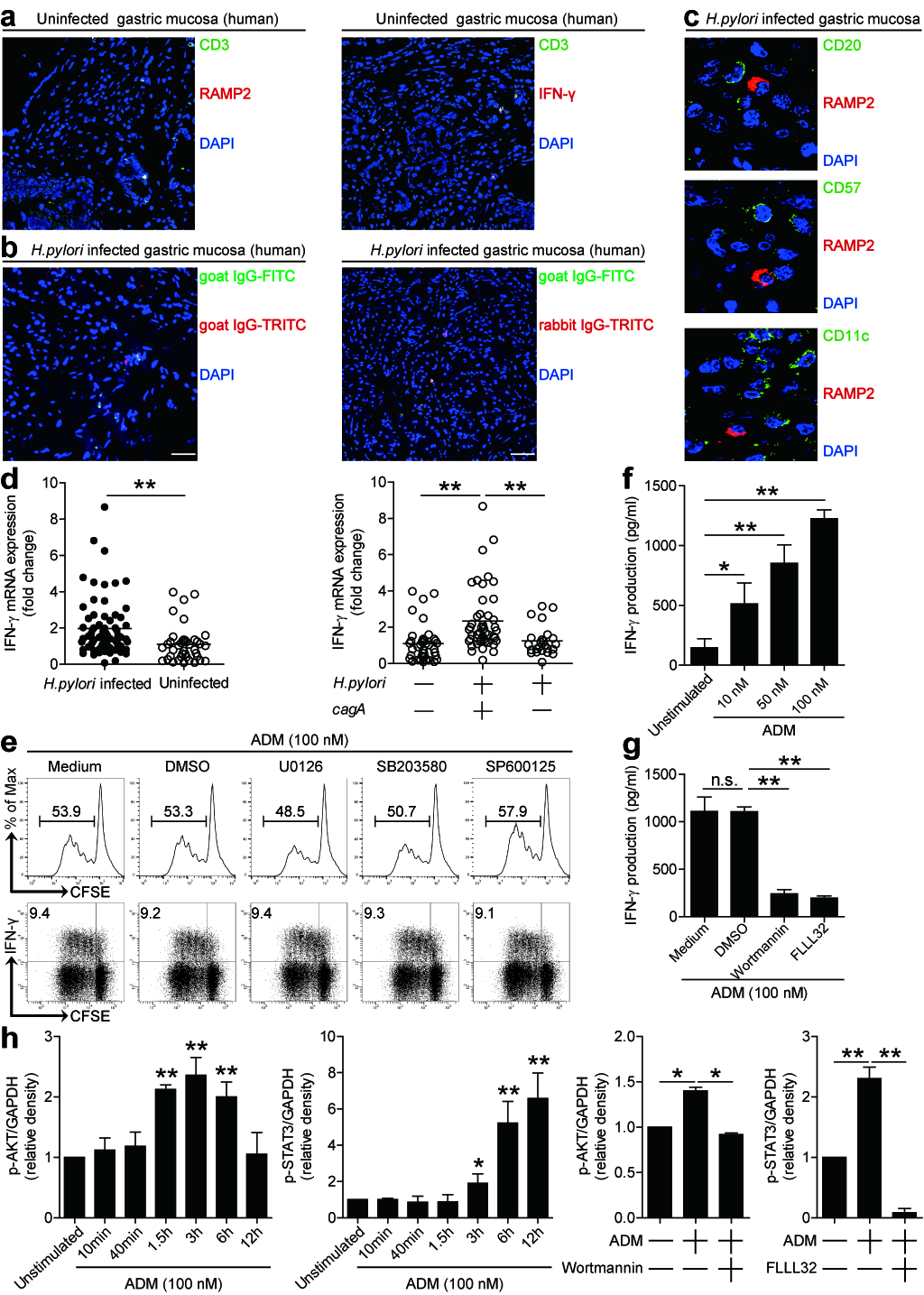

Supplement: Supplementary file 7 — Supplementary Figure 4 [file 41419_2020_2391_MOESM7_ESM.tif]

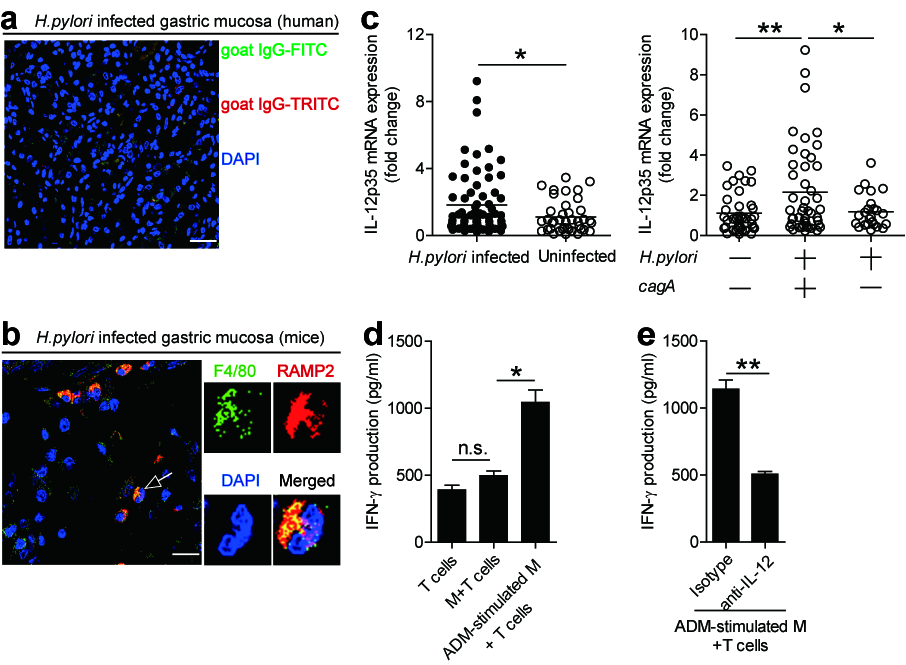

Supplement: Supplementary file 8 — Supplementary Figure 5 [file 41419_2020_2391_MOESM8_ESM.tif]
