# Supplementary material for: Helicobacter pylori-induced adrenomedullin modulates IFN-γ-producing T-cell responses and contributes to gastritis
Source: Cell Death Dis. 2020 Mar 17;11(3):189. doi: 10.1038/s41419-020-2391-6 (PMC7078296; doi:10.1038/s41419-020-2391-6)
Supplement: Supplementary file 9 — Supplementary Figure Legends [file 41419_2020_2391_MOESM9_ESM.doc]

Helicobacter pylori-induced adrenomedullin modulates IFN-γ-producing T-cell responses and contribute to gastritis

**Supplementary Figure Legends**

**Supplementary Figure 1.** *H. pylori* stimulates gastric epithelial cells express ADM. (a) and (b) ADM protein in gastric antrum (a) or gastric corpus (b) of WT *H. pylori*-infected mice on day 21 p.i. was analyzed by immunohistochemical staining. Scale bars: 100 microns. (c) Representative immunofluorescence staining images showing CD45^+^ hematopoietic immune cells (red) and ADM-expressing (green) cells in gastric mucosa of *H. pylori*-infected patients. Scale bars: 50 microns. (d) Representative immunofluorescence staining images showing ADM-expressing (red) and CD326^+^ gastric epithelial cells (green) in gastric mucosa of uninfected donors. Scale bars: 100 microns. (e) Representative immunofluorescence staining images showing ADM-expressing (red) and H^+^/K^+^ ATPase^+^ parietal cells (green) in gastric mucosa of uninfected donors. Scale bars: 100 microns. (f) Representative immunofluorescence staining images showing ADM-expressing (red) and pepsinogen II^+^ chief cells (green) in gastric mucosa of uninfected donors. Scale bars: 100 microns. (g) Secondary antibody-only stained sections as controls for the staining in Figure 2a-c. Scale bars: 100 microns. (h) ADM mRNA expression in *H. pylori* 11637-infected, *H. pylori* 26695-infected and uninfected HGC-27 cells at 12 or 24 h (MOI=100) was analyzed by real-time PCR (n=3). (i) ADM mRNA expression and ADM protein in/from WT *H. pylori*-infected, *ΔcagA*-infected, and uninfected HGC-27 cells (MOI=100, 24 h) were analyzed by real-time PCR and western blot and statistically analyzed (n=3). Results are representative of 3 independent experiments. Protein loading is shown in the coomassie brilliant blue staining gel. The supernatants were used to measure the expression of ADM by western blot and the total cell lysates were used to measure the expression of ADM by real-time PCR. *, *P*<0.05, and **, *P*<0.01 for groups connected by horizontal lines compared.

**Supplementary Figure 2.** *H. pylori* stimulates gastric epithelial cells to express ADM via PI3K-AKT pathway. AGS cells were pre-treated with signal pathway inhibitors and then stimulated with WT *H. pylori* (MOI=100) for 24 h. ADM mRNA expression in AGS cells was compared (n=3). ***P*<0.01, and n.s. *P*>0.05 for groups connected by horizontal lines compared.

**Supplementary Figure 3.** *In vivo* blockade of ADM significantly reduced inflammation and IFN-γ-producing T-cell responses in the stomach during *H. pylori* infection. (a) The levels of Ly6G^-^CD11b^+^ monocytes, Ly6G^+^CD11b^+^ neutrophils, NK1.1^+^ natural killer cells (NK cells), CD19^+^ B cells, IL-4^+^ T cells, and IL-17^+^ T cells in gastric mucosa of WT *H. pylori*-infected mice injected with Abs against ADM or corresponding control IgG on day 21 p.i. were compared (n=5). (b) The numbers of CD3^+^ T cells in gastric mucosa of WT *H. pylori*-infected mice injected with Abs against ADM or corresponding control IgG on day 21 p.i. were compared (n=5). (c) The IFN-γ-producing T-cell responses in peyer’s patches of WT *H. pylori*-infected mice injected with Abs against ADM or corresponding control IgG on day 21 p.i. were compared (n=5). (d) The mucosa thickness in gastric mucosa of WT *H. pylori*-infected mice injected with Abs against ADM or corresponding control IgG on day 21 p.i. were compared (n=5). n.s. *P*>0.05 for groups connected by horizontal lines compared.

**Supplementary Figure 4.** ADM promotes IFN-γ-producing T-cell responses via PI3K-AKT and STAT3 activation. (a) Representative immunofluorescence staining images showing RAMP2-expressing (red) CD3^+^ T cells (green) and IFN-γ-expressing (red) CD3^+^ T cells (green) in gastric mucosa of uninfected donors. Scale bars: 50 microns. (b) Secondary antibody-only stained sections as controls for the stainings in Figure 5A. Scale bars: 20 microns (left), 100 microns (right). (c) Representative immunofluorescence staining images showing RAMP2-expressing (red) cells and CD20^+^ B cells (green), RAMP2-expressing (red) cells and CD57^+^ NK cells (green), RAMP2-expressing (red) cells and CD11c^+^ dendritic cells (green) in gastric mucosa of *H. pylori*-infected patients. (d) IFN-γ mRNA expression in gastric mucosa of uninfected donors (n=40) and *H. pylori*-infected patients (n=70) was compared. IFN-γ mRNA expression in gastric mucosa of uninfected donors (n=40), *cagA*^-^ *H. pylori*-infected (n=24), and *cagA*^+^ *H. pylori*-infected (n=46) patients was compared. (e) T cells were pre-treated with signal pathway inhibitors and then stimulated **with ADM (100 nM) for 5 days** as described in Methods**.** T cell proliferation and IFN-γ production was assessed **by** flow cytometry. Results are representative of 3 independent experiments. (f) and (g) T cells were stimulated **with ADM** (10, 50, 100 nM) (f), or pre-treated with Wortmannin (a PI3K-AKT inhibitor) and then stimulated **with ADM (100 nM) (g) for 5 days** as described in Methods**.** The IFN-γ production from T-cell culture was detected **by** ELISA and statistically analyzed (n=3). (h) Statistically analyze of western blot of Figure 5f (n=3). Results are representative of 3 independent experiments. **P*<0.05, ***P*<0.01, and n.s. *P*>0.05 for groups connected by horizontal lines compared, or compared with uninfected cells.

**Supplementary Figure 5.** ADM-stimulated macrophages induce IFN-γ-producing T-cell responses. (a) Secondary antibody-only stained sections as controls for the staining in Figure 6a. Scale bars: 50 microns. (b) Representative immunofluorescence staining images showing RAMP2-expressing (red) F4/80^+^ macrophages (green) in gastric mucosa of WT *H. pylori*-infected mice on day 21 p.i.. Scale bars: 20 microns. (c) IL-12p35 mRNA expression in gastric mucosa of uninfected donors (n=40) and *H. pylori*-infected patients (n=70) was compared. IL-12p35 mRNA expression in gastric mucosa of uninfected donors (n=40), *cagA*^-^ *H. pylori*-infected (n=24), and *cagA*^+^ *H. pylori*-infected (n=46) patients was compared. (d) and (e) The IFN-γ production from T cell-macrophage co-culture was detected **by** ELISA **as described in Methods** and statistically analyzed (n=3). **P*<0.05, ***P*<0.01, and n.s., *P*>0.05 for groups connected by horizontal lines compared.
